# Environmental risk and market approval for human pharmaceuticals

**DOI:** 10.1007/s40592-024-00195-1

**Published:** 2024-07-03

**Authors:** Davide Fumagalli

**Affiliations:** Department Philosophy, Linguistics and Theory of Science, University of Gothenburg, Renstromsgatan 6, 412 55 Gothenburg, Sweden

**Keywords:** Environmental policy, Pharmaceutical pollution, Antibiotics, Antimicrobial resistance, Ethics

## Abstract

This paper contributes to the growing discussion about how to mitigate pharmaceutical pollution, which is a threat to human, animal, and environmental health as well as a potential driver of antimicrobial resistance. It identifies market approval of pharmaceuticals as one of the most powerful ways to shape producer behavior and highlights that applying this tool raises ethical issues given that it might impact patients’ access to medicines. The paper identifies seven different policy options that progressively give environmental considerations increased priority in the approval process, identifies ethically relevant interests affected by such policies, and makes explicit tensions and necessary tradeoffs between these interests. While arguing that the current European regulation gives insufficient weight to environmental considerations, the paper highlights concerns with the strongest policy options, on the grounds that these may very well endanger patients’ access to effective medication.

## Introduction

Institutions and researchers have recently identified environmental pollution from the use and production of medicines as a threat to human and environmental health (Beek et al. 2016; Mackuľak et al. 2019; UNEP 2019; European Commission 2020; European Parliament 2020; WHO/FAO/IOE 2020). For instance, studies have demonstrated that the release of Diclofenac is harmful to aquatic ecosystems, potentially resulting in adverse effects on wildlife. These effects include organ damage and diminished hatching success in fish, neurotoxicity and oxidative stress in molluscs, and hormonal disruptions in frogs (Islas-Flores et al. 2019). Moreover, the release of antibiotics in the environment may increase the selection pressure on bacteria which promotes the mobilization and horizontal transfer of resistance genes (Larsson and Flach 2021) in bacteria. This phenomenon contributes significantly to the problem of antimicrobial (AMR) resistance, which represents a gradual erosion of the efficacy of antimicrobial drugs. Effective antibiotics are indispensable for the treatment of bacterial infections and are fundamental for numerous medical interventions, particularly surgical procedures, which in many cases would become substantially more dangerous without access to antibiotic treatment (Laxminarayan et al. 2013; O’Neill 2016).

These concerns emphasize the necessity of regulating and reducing environmental pollution caused by pharmaceuticals, addressing the potential hazards that it poses to human, animal and environmental health. Various approaches can be employed to achieve this goal, involving different actors and stakeholders (Nijsingh et al. 2019; Malmqvist et al. 2023). Malmqvist and Munthe (2020) have identified four areas where new regulations can be introduced to encourage the pharmaceutical industry to adopt more sustainable practices in drug production and development: authorization, procurement, public subsidy, and generic substitution of drugs. In light of recent initiatives within the EU to enhance the consideration of environmental risk at the authorization level (European Parliament 2019; European Commission 2023), this paper will explore various approaches in this area and analyze the tradeoffs between different normatively significant interests that these require.^1^ Interventions that regulate the market approval process can be extremely impactful, as the threat of rejection of the application can be used to apply substantial pressure on the industry, however, it also carries exceptional risk of endangering access to new medication (Nijsingh et al. 2019). For these reasons, a good deal of precaution is necessary (Malmqvist and Munthe 2020).

Against this background, the present paper will address the following question: How should the consideration for environmental risk affect the market authorization process for human pharmaceuticals? The paper is organized as follows. Section 2 outlines the current legislation for the drug approval process for the EU market, highlighting the asymmetries between the requirements for human and veterinary pharmaceuticals. Section 3 makes a normative case in favour of the introduction of environmental concerns in the market approval process for human pharmaceuticals, identifies ethically relevant but potentially conflicting interests that would be affected by changes in the approval process and presents alternative policy models that balance these interests in different ways. Section 4 discusses which model or models represent an ethically defensible trade-off between the competing interests.

## Current legislation

Currently, the requirements for market authorization for human and animal pharmaceuticals are outlined in several directives.^2^ These directives require an Environmental Risk Assessment (ERA) as a part of the market application for a product. An ERA comprises a series of increasingly detailed and tiered tests which assess the potential impact that a drug could have on the environment and its potential risks (Lee and Choi 2019). The assessment of the quality of the data submitted by the producers is carried out by the Committee for Medicinal Products for Human Use (CHMP), a subsidiary of the European Medicines Agency (EMA). Alternative tests may be accepted, provided that they are appropriately justified in the ERA report and that testing methods are deemed fit for the purpose.

While major awareness of pharmaceutical pollution was already present in the 2001’s Directive, it is more strongly emphasized in the recent ﻿European Union’s Strategic Approach to Pharmaceuticals in the Environment, which aims to address the problem more concretely (European Parliament and Council of the European Union 2019b/6; European Parliament and Council of the European Union 2020/741). The European Commission is currently working on a new directive that targets, among other things, the quality and consistency of assessment of the environmental risks of human pharmaceuticals (European Commission 2023).

### Human pharmaceuticals

The EMA released some guidelines in 2006 (European Medicines Agency 2006), which build on the 2001 Directive. According to the guidelines and the 2001 Directive, for a medicine to be accepted on the market, the producer needs to submit a clinical benefit-risk assessment (BRA) that demonstrates that the benefits of the drug for patients outweigh the risks to their health. The approval of an application depends only on the clinical benefit-risk evaluation and not on the ERA. Thus, the main aspect that is analysed when determining whether to approve a medicine is its impact on patients in the target population and not on its potential broader environmental impact (or related indirect consequences for other patients).

EMA writes:

Evaluation of the potential environmental risks posed by the medicinal product. This impact shall be assessed and, on a case-by-case basis, specific arrangements to limit it shall be envisaged. In any event this impact should not constitute a criterion for refusal of a marketing authorization (European Medicines Agency 2006, p. 3).

While the environmental impact of pharmaceuticals is clearly acknowledged already in the 2001’s Directive, market approval is never conditional to it. Rather, any environmental side effect of a drug are to be handled after approval, for instance by taking precautions when disposing of unused medicines or medical waste (EMA 2006). Additionally, the European Parliament and Council suggested preventive measures such as increased awareness, promotion of prudent use of pharmaceuticals, and increased transparency during the manufacturing process (European Commission and Council of the European Union 2019/6). These measures attempt to limit pharmaceutical pollution in the environment, without fundamentally changing the market access conditions. The new Directive proposal currently under examination significantly increases the importance assigned to environmental risk by requiring a complete ERA along with a list of possible risk mitigation measures targeting harms identified in the ERA:

(70) Marketing authorisation applications for medicinal products in the Union should include an Environmental Risk Assessment (ERA) and risk mitigation measures. If the applicant fails to submit a complete or sufficiently substantiated environmental risk assessment or they do not propose risk mitigation measures to sufficiently address the risks identified in the environmental risk assessment, the marketing authorisation should be refused. The ERA should be updated when new data or knowledge about relevant risks becomes available (European Commission 2023, pp. 30–31).

That said, within the legislation currently in place, the environmental impact of a human pharmaceutical does not affect its approval, and any environment-related risk is handled by preventive or remedial practices after the drug is approved.^3^ Normatively speaking, the clinical benefit-risk analysis is the only aspect that is relevant for market applications for human pharmaceuticals.

### Veterinary pharmaceuticals

The European Parliament and European Council adopted the current Regulation in December 2018 and it took effect on 28 January 2022 as Regulation EU/6 (European Parliament and of the Council of the European Union 2018).^4^

In the Regulation’s *Article 4, Definitions*, the notion of ‘benefit-risk balance’ is given a broader definition than in the case of human pharmaceuticals. Specifically, the risks that are relevant are of three kinds (European Parliament and Council of the European Union 2019b, p. 5):

(a) any risk relating to the quality, safety and efficacy of the veterinary medicinal products as regards animal or human health;

(b) any risk of undesirable effects on the environment;

(c) any risk relating to the development of resistance;

Moreover, according to *Article 37,* it is possible to deny the market authorization if (European Parliament and Council of the European Union 2019b, pp. 26–27):

(b) benefit-risk balance of the veterinary product is negative.

[…]

(f) risk for public health in case of development of antimicrobial resistance or antiparasitic resistance outweighs the benefits of the veterinary medicinal product to animal health;

(i) risks to public or animal health or to the environment is not sufficiently addressed

By contrast to the case of human-use pharmaceuticals, the approval of a veterinary drug is thus not simply determined by the clinical benefit-risk assessment. A drug might safely and effectively treat, say, an infection in a farm animal, but its approval could be denied because of its environmental effects such as driving antimicrobial resistance.

### Comparison of legislation for human and veterinary pharmaceuticals

There is thus a conspicuous asymmetry regarding the relevance of pharmaceutical pollution for the approval of human and veterinary pharmaceuticals. Awareness about the risks posed by such pollution has had a significant impact on veterinary regulation, but less so in the case of human pharmaceuticals. In no circumstances can market authorization for a human drug be denied on the base of its environmental impact. For human drugs, any level of environmental risk is justified if the use of a pharmaceutical provides a sufficiently high benefit-to-risk ratio for a human patient. In practice, this entails that the interests of patients in the target population are given a higher priority than other interests affected by the drug approval process, be they related to the environment, animal health, public health, or future patients. This enables the approval of clinically safe and effective, but potentially extremely environmentally hazardous human drugs, and only allows action to be taken to address such hazards once a drug is on the market. The directive for human pharmaceuticals being currently discussed (European Commission 2023) would simply refuse the market application of a similar product, whenever the mitigation measures would prove insufficient. As for veterinary medicines, approval can be denied in order to prevent danger to human health or interests, for instance in the form of antiparasitic or antimicrobial resistance. In this context then, the interest of animals in a drug’s target population can be subordinated to the protection of human or environmental health. This is indicative of different levels of normative priority assigned to patients' access to pharmaceuticals and protection from environmental risk. The following section will make the case for including consideration of environmental risk in the approval process of human pharmaceuticals, outline alternative policy options for doing so, along with an analysis of how they would impact different morally relevant interests.

## Including consideration of environmental risk in the approval process for human pharmaceuticals

As shown in the preceding section, the environmental risk of a human pharmaceutical has no relevance to its approval on the market. This can lead to the approval of drugs that are extremely polluting and contribute to other drugs losing their efficacy, as in the case of AMR. This is potentially problematic because such pollution can have a significant impact on human health, as well as wildlife and the environment.

Several prima facie ethical reasons can be given in favour of the introduction of environmental considerations in the market approval process for human pharmaceuticals. The first reason is preventing direct potential harm to human health stemming from pharmaceutical pollution (European Commission 2019; Mackuľak et al. 2019; Batucan et al. 2022). Pharmaceuticals contain active pharmaceutical ingredients (APIs), which have stable molecular structures making them particularly resilient in the environment. APIs can stay in the environment for a long time, and have in some cases been shown to induce serious biological changes in local fauna, particularly in certain environments e.g. close to pharmaceuticals manufacturing sites, sewage and water treatment plants, and aquaculture sites ( Love et al. 2011; Gaw et al. 2014; Lonappan et al. 2016; Sathishkumar et al. 2020). There is a concern that the release of APIs and pharmaceutical byproducts of the manufacturing process might have similar impact on human beings. In particular, Okeke et al. (2022) have highlighted the risk of bioaccumulation of pharmaceutical through drinking water or food(i.e. fish, vegetables, fruits), endangering in particular already immunocompromised individuals, by inducing metabolic disorders or cancer.

A second prima facie reason is prevention of indirect harm resulting from pharmaceutical pollution-induced AMR, which represents a substantial threat to the functioning of healthcare systems (Littmann and Viens 2015; Walsh et al. 2023). Recent research pointed out the specific role of antibiotic use, production and excretion in driving resistance by increasing the selection pressure for relevant groups of bacteria in selected environments such as wastewater treatment plants (Larsson and Flach 2021). Higher levels of AMR mean that antimicrobial drugs are becoming less effective which in turn implies that untreated infections become substantially more threatening. One should not disregard that antibiotics have a key role in enabling safe and more complex medical interventions like surgeries or chemotherapy and staving off neonatal infections (Russell et al. 2023).

A third, related reason is that AMR threatens long-term access to effective medications for future generations, making it a problem of intergenerational justice (Barry 1997; Green 1977) and sustainability (Munthe et al. 2021).

Finally, a fourth reason concerns intragenerational justice related to unequal healthcare access (Sen 2002; Temkin 2000, 2003; Reid 2020). In particular, researchers have highlighted how poor and vulnerable populations are disproportionally threatened by AMR because of limited access to medicines and greater susceptibility to infections with resistant bacteria (Selgelid 2007). Additionally, inequality in healthcare access may compound AMR since poorly treated infections may contribute to the selection of resistance genes (Millar 2020).

Preventing pharmaceutical pollution through the market approval process specifically (rather than through other interventions) can be justified by appealing to effectiveness: since authorization agencies can determine what drugs can be sold on a market, they have significant power to influence pharmaceutical companies’ behavior (Nijsingh et al. 2020). That being said, given the wide impact that such an intervention might have in terms of, for example, what type of drugs will be produced, and how fast will they be produced, balancing out the effectiveness of the intervention against the needs of patients to access effective mediation becomes crucial.

The remainder of this section outlines different options for including environmental concerns in the approval process of human pharmaceuticals, focusing on policies that are currently being introduced in the EU or that have been suggested in recent academic literature. As will become clear, such policies face a fundamental tension between individual and collective interest of the sort that is familiar from debates in public health ethics and political philosophy.^5^ Specifically, they require tradeoffs between long-term public health considerations and short-term clinical ethical considerations regarding benefits and safety for patients in care (Nijsingh et al. 2019).

Nijsingh et al. (2019) have identified many different stakeholders whose interests may be affected by attempts to address pharmaceutical pollution (including drug companies, physicians and other healthcare staff, as well as patients, animals and the environment). I will focus on those interests that are already of central concern in the market approval process, namely, human health-related ones. In this strongly anthropocentric context, the presumption is that protecting and promoting such interests is the main reason to even have pharmaceutical regulation and risk assessment in the first place. From a normative standpoint in the legislative framework I am considering, the interests of other kinds, such as animals or the environment, are not considered in their own right but only insofar as they indirectly impact human health interests. Undoubtedly lowering pharmaceutical pollution may potentially positively impact wild animals and environmental entities such as ecosystems. However, given that the amount of normative weight that ought to be assigned to the interests of non-human animals and environmental entities is highly controversial I will not focus on them here (Singer 1990; O’Neill 1997; Kamenshchikova et al. 2021;). For example then, the acknowledged environmental impact of drugs like Diclofenac is only to be considered as long as it is potentially harmful to human health (Sathishkumar et al. 2020; Batucan et al. 2022).

Among human health-related interests, three types stand out as significant:

The interest of individuals whose direct access to medication would be hampered in case a drug was rejected or delayed in production for environmental reasons. I will refer to this interest using the term *direct patient access*.

The interest of individuals whose health would be endangered by the approval of environmentally hazardous drugs. This group of people includes patients who could experience increased health risk, healthy people which might become ill because of environmental pollution, and patients negatively affected by environmentally-driven drug resistance. I will refer to this interest using the term *environmental protection*.

The interest of individuals whose health may be endangered because of market changes, such as increased price, because of new regulation. I will refer to this interest using the term *indirect patient access*.

**Fig. 1:**
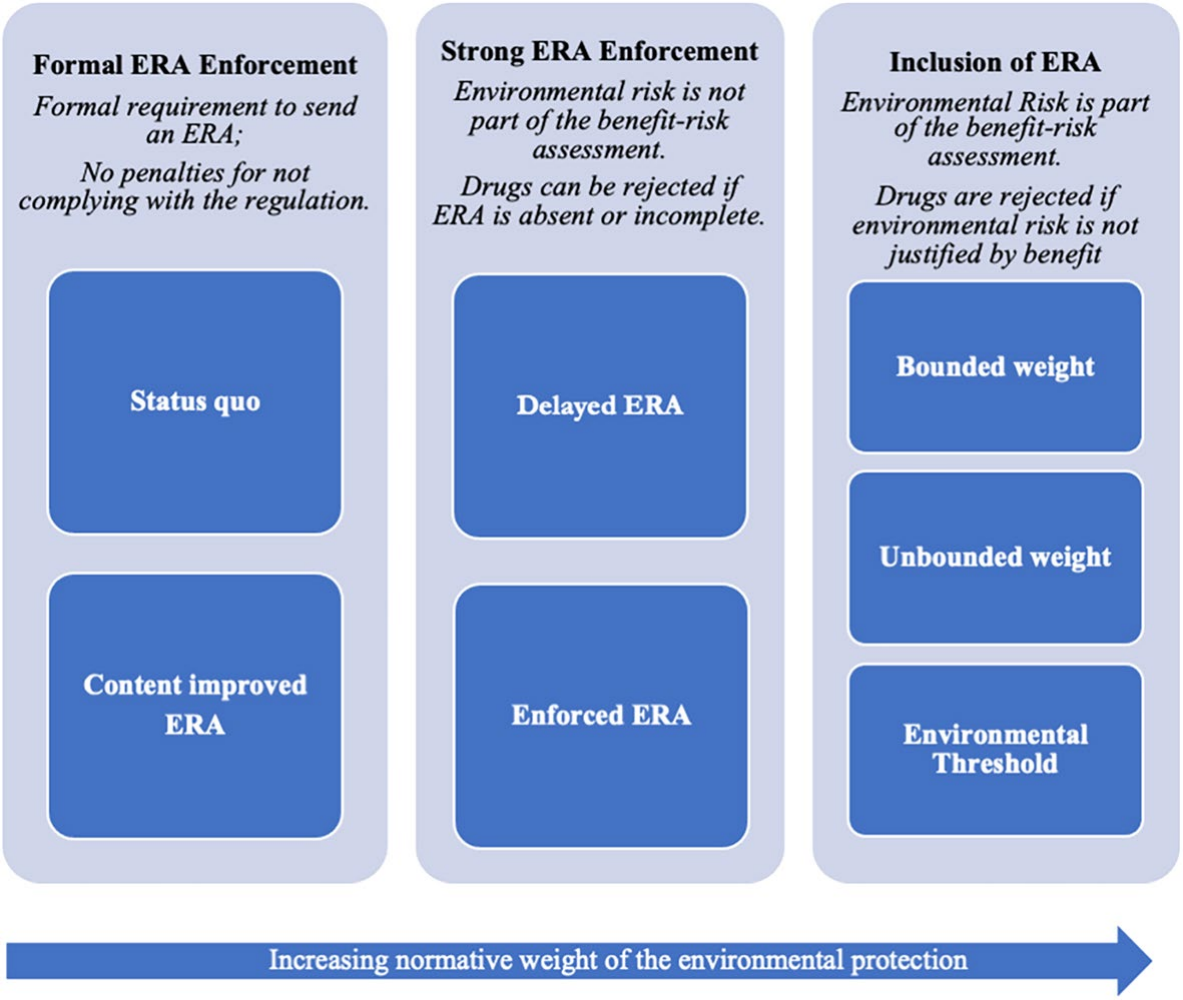
The figure graphically presents the three categories of policy models. From left to right, the priority given to environmental protection gets increasingly stronger and related requirements get increasingly stricter

For the sake of clarity, the remainder of this section will present different policy options that represent different priorities between the first two of these interests, direct patient access and environmental protection, whereas the third kind of interests, indirect patient access, will be analysed separately at the end of this section. I will draw on recent policy suggestions and the literature on pharmaceutical pollution to distinguish seven normatively different models that can be grouped into three categories (See Fig. 1). The models are ordered by how they assign progressively more priority to environmental protection. The first category does not consider the ERA, nor its content, to be relevant to whether a drug is approved; the models included in this category are *Status quo* and *Content improved ERA*. The second category introduces strict and enforced requirements regarding the submission of the ERA, but not its content; this includes *Delayed ERA* and *Enforced ERA*. The third category considers the submission of the ERA strictly compulsory and considers the level of environmental risk posed by a drug as potentially relevant for approval (Malmqvist and Munthe 2020); this includes the *Bounded weight*, *Unbounded weight* and the *Environmental Threshold* model. Under no condition will the first four frameworks make it possible to refuse a market application or argue for market withdrawal only on the basis of high environmental risk. The last three, on the other hand, will allow the rejection of a market application although in different ways and in different circumstances.

### Formal ERA enforcement

#### Status quo

This model is the one currently in use in the EU. Formally it requires a producer to include an ERA when submitting a market application for a drug. In accordance with Directive 2001/83/EC as amended, the ‘environmental impact should be assessed, and on a case-by-case basis, specific arrangements to limit the impact should be considered’. However, in any event, ‘this impact should not constitute a criterion for refusal of a marketing authorization for medicinal products for human use’ (EMA 2006, p. 3).

﻿﻿Caneva et al. (2014) point out that the absence of penalties for not complying with the regulation makes it possible to receive market access without submitting an ERA. Moreover, even when the ERA is submitted, ﻿they highlight how the requested studies were either ‘missing or of unsatisfactory quality for 83%’ of the total (Caneva et al. 2014, p. 314).

As it currently stands, this framework does present a strong issue of compliance and enforcement, as it leaves the option of not submitting an ERA open in practice, limiting the opportunity to address environmental risk after a drug is approved. As such, this framework is the one that prioritizes environmental health the least of the ones I consider here.

#### Content improved ERA

This framework introduces additional components to the content of the ERA, whilst not making its submission any more compulsory or introducing any time constraint. As an example, Ågerstrand et al. (2015) recommend improving EMA’s control over the environmental risk assessment of pharmaceuticals for human use by including more detailed information, for instance by indicating the risk for the development of antibiotic resistance or including an ecotoxicological assessment. An additional suggestion is the inclusion of data relative to the production of active pharmaceutical ingredients(API) for the medicine. Similar suggestions are made by Lee and Choi (2019). These solutions would in theory allow to increase transparency, and gain important data regarding pharmaceutical pollution, which in the long term can help to establish new more effective risk mitigation practices.

Regardless of which specific content the ERA will require in the renewed legislation, however, the previous considerations regarding compliance and enforcement will remain. Thus, while mandating a more complete ERA would likely serve environmental protection slightly more than in *Status Quo*, patient access would still likely be normatively more relevant.

### Increased requirements for the submission of the ERA

#### Delayed ERA

A first, not very stringent, requirement can be introduced by requiring an ERA but allowing delayed submission of it. As such, the authorization assessment can start in the absence of an ERA, but will not be concluded without it. Approving a safe and effective but highly polluting drug will still be possible, but at least there will be more awareness and transparency about the environmental impact.

The main advantage of this solution consists in providing additional time for producers to provide the ERA without causing a delay in access that would hinder timely patient access. Doing this makes it possible to have a more complete picture of the environmental impact related to drug manufacturing thanks to ERAs, as well as to give valuable data for the environmental research (O’Neill 2016), keeping priority on patient access. Like with the currently used framework, any meaningful environmental side effect would be handled case-by-case by measures applied after approval. The degree to which environmental pollution would be mitigated would depend on what these measures are.

This option imposes stricter requirements for the recording of environmental risks than the previously outlined frameworks. As such, it does protect environmental health more effectively than the *Status quo* and *Content improved ERA* frameworks, but to a lesser extent than the options I will examine next.

#### Enforced ERA

A second, more constraining approach is to make the submission of an ERA compulsory for approval on the market; with this model, the option of noncompliance regarding the submission of the ERA is removed. This type of solution was already adopted in Sweden (Strategi för Sveriges arbete för en giftfri miljö 2012), and further suggested by Ågerstrand et al. (2015). This solution could have a positive effect of encouraging transparency related to the manufacturing process and facilitating any post-approval mitigation measures. As seen above, such effects can also be secured by additional rules on what type of information the ERA should contain (*Content improved ERA*), and the two models may well be combined. Note that in this framework, the specific content of the ERA would still not impact whether the drug is approved. It would be possible to accept on the market an extremely environmentally harmful drug, so long as the ERA study was submitted.

In this scenario, the ERA’s content itself is still not considered sufficient to refuse a market application. However, improved transparency and labelling could incentivize the pharmaceutical industry to ensure stronger control of pollution from their production (O’Neill 2016).

Unlike in the previous framework, in this case, there would be no conditional approval. The submission of the ERA would be necessary at the time of approval and follow a hard deadline. By introducing the presence of an ERA as a stringent condition for market access, this framework would make available additional environmental data which could be valuable for developing more effective post-approval remedial actions. However, such a requirement would also require producers to invest more time and resources in the application for market approval than at present, which could potentially slow down the introduction of new drugs on the market, as well as possibly scaring producers off the market. This means that of the first three frameworks presented, this one prioritizes direct patient access the least and environmental health the most.

### Environmental risk as a factor for market approval

#### Bounded weight

This framework represents the first, cautious, way to include the environmental risk of a pharmaceutical in the benefit-risk assessment of human medicines. Whether or not a drug is accepted on the market will, in certain cases, depend on how the environmental impact of the drug and clinical efficacy will compare to each other. This approach has been proposed by Ågerstrand et al. (2015, p. 5341):

The ERA should be included in the risk-benefit analysis, but…[t]he possibility to consider the environmental aspects in the authorization should only aim at denying further approvals of products containing a known problematic API when there are already products on the market that cover the specific medical need to the same extent though associated with a lower environmental risk.

This model offers the first example of a scenario where the rejection of a drug could be motivated by its environmental impact. With this framework, it would be possible to refuse a market application if the new product has a worse environmental impact than an already marketed drug that addresses a similar clinical need. In theory, this model forces the market to produce drugs that are more environmentally sustainable than the ones already in use, without critically hampering patient access to more effective drugs.

However, this framework might not be easily applicable in many situations. Suppose that two drugs, *x* and *y*, have different APIs. From a clinical point of view, it is unlikely that *x* and *y* will have identical clinical effects for identical patient populations. They will likely vary at least somewhat with respect to effect and side-effects or be indicated for at least somewhat different populations. Hence, it is going to be quite hard to find two clinically equivalent drugs with different APIs that address the exact same medical need (Meredith 2009; Tothfalusi 2008). So long as a drug targets a slightly different patient population or has slightly greater effect or lower risk for an identical population than a drug already on the market, it would be possible for it to gain market access independently of environmental concerns.

In practice, the performance of this model would largely depend on how the relevant medical needs are defined. If they are defined narrowly, it will not be possible to deny authorization very often, as already noted. But in cases where market authorization *is* denied there would presumably be no negative health impact on patients, whose needs are already fulfilled. On the contrary, if the need is defined more broadly, it will be easier to find scenarios where comparable drugs can be already found on the market. This would mean that refusing market access on grounds of environmental risk would be more common, but the risk of at least marginally sacrificing patients’ interest in safe and effective medicines would be greater.^6^ Thus, the interests of environmental health will receive higher priority the more loosely the relevant needs are defined.

Whichever of the two interpretations is applied, this framework allows refusing a market application on an environmental basis, albeit in a limited number of cases. As such, this framework prioritizes the interests of environmental health more than the previous three frameworks I have considered.

#### Unbounded weight model

This framework is similar to the previous one because it relies on including the environmental impact of a human pharmaceutical in the benefit-risk assessment. In this model, the refusal of an application on an environmental basis can be justified, even in absence of the availability of another clinically equally good drug. This would be possible when the environmental risk (together with clinical risks) are taken to outweigh the clinical benefits. However, this scenario would leave open the possibility of ensuring patient access to clinically valuable drugs despite adverse environmental effects. The impact of this model will depend on *how much* weight is given to clinical risk, clinical benefit, and environmental risk, respectively (Nijsingh et al. 2019; Malmqvist & Munthe 2020). However, it would in principle prioritize environmental protection more strongly than the previously considered models by, for example, allowing the rejection of a drug that would significantly drive AMR.

#### Environmental threshold

In the last scenario, a clinically effective drug is rejected if its environmental impact is above a certain threshold. Within this framework, it will be possible to reject a market application solely by virtue of its environmental effects, even in cases where it might offer revolutionary therapeutical solutions. This is the most drastic option of the ones mentioned until now.

This framework sets a very strict and specific limit to what degree of environmental risk would be tolerable. The implication is that a clinically safe and effective drug might be denied market access because of the set threshold. In theory, this option allows prioritizing the interests of environmental health the most, but this all depends on how much environmental risk is actually tolerated. Some qualifications should be made. On the one hand, if the tolerated environmental impact was very high, even a strict threshold framework might be incapable of refusing market access to very polluting drugs. In this case, patient access would have a higher normative priority. On the other hand, if the tolerated environmental impact was very low, it would be possible to refuse market access even to drugs that have a more average environmental impact. In this case, normative priority would be assigned differently and undeniably to environmental health, at the expense of patient access.

A variant of this *Threshold* framework can be identified in the directive proposed by the European Commission, which introduces a stronger requirement for the submission of a complete ERA, as well as the possibility of refusing a market application if the applicant fails to “propose risk mitigation measures to sufficiently address the risks identified in the environmental risk assessment” (European Commission 2023, p. 32). Despite not openly referencing the notion of an environmental risk threshold, the new directive operates under the assumption that certain levels of environmental risk are not tolerable, and either a drug has an acceptable environmental impact or the producer needs to present sufficient risk mitigation measures.

Both versions of the *Environmental Threshold* model would prioritize the interests of environmental health more than all the options we considered in this section. Compared to the *Bounded* and *Unbounded Weight* framework, this framework is more rigid, and it sets a strict threshold, which in principle provides a powerful instrument for preventing environmental risk but also potentially requires foregoing significant clinical benefits (Nijsingh et al. 2019; Malmqvist & Munthe 2020) by potentially reducing patient access or scaring producers off the market. That said, the tradeoffs between these interests will vary substantially depending on where the threshold is set and how easy it is for producers to comply. It should be noted that in practice there is a limited understanding of what specific set of environmental circumstances enable the emergence of resistance inclinically important bacteria (Larsson and Flach 2021), and of the relevant environmental pathways for the pharmaceuticals or their impact on human health (Wuijts et al. 2017). As such, understanding precisely how and where to set the thresholds for the ERA remains a hard epistemic challenge.

### Indirect patient access and market dynamics

The third interest is represented by individuals whose health may be endangered because of indirect market changes, for instance patients with health conditions that receive fewer resources because of increased drug prices. The reason for discussing this separately is that overall this group has a relatively stable interest in avoiding the introduction of new regulations. New regulations and potentially environmentally safer drugs would likely come with very complex consequences of which three should be noted in particular. The more demanding the new regulation will be, the higher is the chance that these consequences will ensue.

Firstly, environmental concerns might cause fewer drugs to be approved, which in turn might mean less competition on the market and potentially higher prices for medicines. Supposing a fixed budget for a healthcare system, this could reduce the resources available for purchasing medicines and more generally for maintaining equally good care.

Secondly, the new requirements related to production could introduce new costs for manufacturers. The reasons could be different but could be due to delayed sales because of necessary testing or forced internalization of environmental externalities. This would likely imply that the price of medicines for healthcare systems and private use would go up (Nijsingh et al. 2019), presenting a scenario similar to the one mentioned above.

Thirdly, if the regulation's requirements are perceived to be too harsh or economically unpalatable, the producers might stop producing certain drugs, compromising access to medicines.

That said, indirect patient access could also benefit from increased weight given environmental concerns. Firstly, the more demanding and presumably effective the criteria for market access are, the stronger the prevention of environmental hazards, which in turn will reduce the number of resources spent on dealing with the consequences of those environmental side effects in healthcare. This could lead to reduced demand for healthcare, meaning that more rather than less resources per health need would be available, again assuming a fixed budget. Secondly, framework options like *Compulsory ERA* would allow the gathering of environmental data and expedite research on pollution levels, which could meaningfully impact indirect patient access as well.

### Conclusion

This section has made explicit how addressing environmental pharmaceutical pollution within the drug approval process will require trade-offs between direct patient access to drugs, the prevention of harm to humans caused by environmental pollution, and indirect patient access to drugs. The seven frameworks presented assign different priorities to these different interests. Specifically, while *Status Quo* assigns the highest priority to direct and indirect patient access, the other models allow assigning progressively more and more priority to environmental protection.

## Discussion and conclusion

The second section of this paper outlined the current EU legislation for the market approval process and highlighted the differences between the requirements for human and veterinary pharmaceuticals. The third section of this paper presented the ethical arguments in favour of introducing environmental concerns in market approval process of human pharmaceuticals, and presented alternative policy frameworks that balance different morally relevant interests in different ways. This section will discuss which framework or frameworks represent the best trade-off or balance between these interests. It is useful to distinguish two extremes in the prioritization between interests, one strongly favoring the current patients’ access to healthcare or people whose health might be endangered because of potential market changes, and the other one strongly prioritizing prevention of pharmaceutical pollution and long-term access to effective medication.

The first extreme is represented by the current regulatory framework, which gives the highest priority to direct and indirect patient access, with no meaningful concern for environmental protection. The effort put into measures of active prevention against environmentally induced health harm is substantially lower than the one dedicated to direct patient access. Such a difference in priority seems hard to justify for two reasons.

The first reason is the precautionary principle (Gardiner 2006; Manson 2002; Sandin 2004). The precautionary principle, despite having different formulations and interpretations, generally mandates engaging in preventive actions to avoid a future, yet uncertain, serious risk or harm. Pharmaceutical API’s are chemically very stable substances, which means that once dispersed in the environment they are not going to effectively contaminate it for a long time. While the specific risks related to each API are not entirely clear, they may in certain cases prove disruptive. Additionally, in the case of antibiotics being released in the environment, there is a meaningful risk of effectively, and irreversibly, selecting for resistant genes. As noted already, this could contribute significantly to the serious public health threat of AMR.. The current legislation effectively encourages polluting behaviour, since it doesn’t even penalize producers for not submitting an ERA. As such, *Status Quo*, penalizes those producers that engage in time-demanding testing necessary to submit a complete and detailed ERA for their products.

The second reason is about fairness in right to healthcare. Several authors pointed out the role of fairness in the healthcare allocation context (Selgelid 2009; Kass 2001). In this context, if we are assuming health interests to be equal, there would be no proper reason to treat differently the various groups of patients that this paper identified. Treating the groups of health interests differently would then be unfair.

The other extreme is represented by directly including environmental risk in the benefit-risk analysis, as in the last three frameworks outlined in Sect. 3. Intuitively, this can ensure that environmental protection is more equally considered than it currently is. However, these frameworks come with their own limits and drawbacks. Three main reasons can be identified against using this type of framework.

The first reason is the unfair imposition of risk (Cai 2021) on current patients. This objection grounds itself on the well-known epistemic problem of precisely estimating how pharmaceutically induced environmental pollution will translate to future clinical risk (Levy 1998). It would be practically very hard to establish what specific requirement the ERA should have because it is unclear how and which type of pollution will meaningfully impact human health in the long term and how. In other words, some environmental side effects are hard, if not impossible, to predict and it makes more practical sense to address them once they materialize by, for example, intervening on wastewater treatment plants rather than on market approval processes (Larsson et al. 2018). Additionally, the production chain is almost oblivious to these issues and any strict changes in the market access requirements will be very demanding to adapt to. This uncertain long-term benefit, obtained by preventing pollution and eventual resulting health risks, is to be contrasted with the clear and established health benefits for current patients, resulting from the standardized methodology for the evaluation of clinical risk in the current legislation. In essence the current patients would be directly paying the price of precaution, for a risk that has not verified yet, and that its particularly hard to pinpoint.

The second reason is the avoidance of harm resulting from pharmaceutical pollution and, specifically, from the intrinsic challenge posed by AMR (Littmann and Viens 2015). Consistent patient access to antibiotics, through use and production, is in itself one of the biggest drivers for bacteria becoming resistant. In other words, the mere fact that antibiotics are used now diminishes the effectiveness of future medication with that same class of antibiotics. Any patient at risk of bacterial infections or in need of a medical procedure presupposing antibiotics has a strong interest in accessing to these drugs. Changing the market approval process will likely slow down the introduction of new antibiotics on the market, in exchange for AMR rising more slowly in the long run. A regulation that prioritizes antibiotic efficacy in the long term with such a strong intervention, carries a strong risk of compromising the current access to effective drugs.

The third reason against this type of intervention is the prevention of harm (Kass 2008) caused by lack of access to new or effective medication. As mentioned in Sect. 3.4, including environmental risk in the benefit-risk analysis is one of the strongest and most impactful ways to affect producer behavior. However, such interventions may also have dynamic effects in the form of slower drug development and increased costs of production resulting in higher drug prices and potentially even in producers leaving the market. Compared to the other frameworks, the introduction of environmental risk directly in the benefit-risk analysis carries a greater risk of such effects because this type of regulation is particularly costly or demanding to comply with. These effects make this type of intervention riskier from the point of view of securing patients’ access to effective medication.

That said, these considerations ignore phenomena such as cross-resistance, extended cross-resistance, and co-resistance.^7^ These mechanisms might speed up the spread of resistant genes and diffuse resistance to more than one antibiotic class. Additionally, recent research suggests that some pharmaceuticals, and not only antibiotics, might effectively select resistant bacteria(Wang et al. 2020) in the environment. This would be extremely problematic as it would spread resistance substantially faster and more diffusely. Monitoring and perhaps limiting the development of cross and co-resistance would be an efficient way to prioritize the long-term availability of antibiotics more fairly, without prejudicing current patient access excessively.

Good reasons can be given in favor of abandoning the current policy model in order to protect environmental health to a greater degree. However, it should be noted that the policy options that giving the greatest importance to such protection, in particular the *Environmental Threshold* model, could have a disruptive impact on direct and indirect patient access to medicines. The goal of protecting long-term human health by addressing environmental risk in the market approval process can be advanced in less disruptive ways by implementing more moderate interventions such as *Delayed ERA* and *Enforced ERA*.

## Footnotes

1 In this context, the terms ‘environmental risk’ and ‘environmental impact’ connote any risk to or impact on human health caused by a chemical substance or a pollutant released in the environment.

2 Directive 2001/84/EC of the European Parliament and of the Council of 6 November 2001 on the Community code relating to medicinal products for human use(European Parliament and Council of the European Union 2001); Directive 92/18/EEC of 20 March 1992 modifying the Annex to Council Directive 81/852/EEC on the approximation of the laws of Member States relating to analytical, pharmacotoxicological and clinical standards and protocols in respect of the texting of veterinary medicinal products; Directive 2004/10/EC of the European Parliament and of the Council of 11 February 2004 on the harmonisation of laws, regulations and administrative provisions relating to the application of the principles of good laboratory practice and the verification of their applications for tests on chemical substances.

3 These might include careful waste and disposal handling of the pharmaceuticals, the reduction and change of packaging, or particular attention to wastewater treatment (Ågerstrand et al. 2015).

4 Regulation (EU) 2019/5 of the European Parliament and of the Council of 11 December 2018 amending Regulation (EC) No 726/2004 laying down Community procedures for the authorisation and supervision of medicinal products for human and veterinary use and establishing a European Medicines Agency, Regulation (EC) No 1901/2006 on medicinal products for paediatric use and Directive 2001/83/EC on the Community code relating to medicinal products for human use (European Parliament and Council of the European Union 2019a).

5 In the context of AMR, see Giubilini (2017) and (Anomaly 2009). In the context of disease control and ﻿justifications for compulsory interventions, see ﻿Verweij (2011) and ﻿Carlsson et al. (2019). For more general discussions about distributive justice and individual rights in public health scenarios, ﻿including for example mandatory treatment and screening, see Persad (2019), Kass (2001) and Dawson (2011).

6 A similar objection is standardly raised against Cost minimization analysis in pharmacoeconomics (Rai and Goyal 2018).

7 Cross-resistance is resistance to all the antibiotics belonging to the same class. Drugs assigned to the same class have the same target of action in the cell, and as such can encourage cross-resistance. Extended Cross-resistance is a type of cross-resistance that can confer resistance to more than one drug class. In practice, a class of antibiotics can select for resistance to other drug classes. This phenomenon occurs even with non-medical products such as the increasingly used household detergents, which can select for multiresistant bacteria. Co-resistance occurs when the member of one drug class co-selects resistance to another class of antibiotics with a totally distinct mode of action (Périchon et al. 2019).
